# LncRNA-XIST Promotes Proliferation and Migration in ox-LDL Stimulated Vascular Smooth Muscle Cells through miR-539-5p/SPP1 Axis

**DOI:** 10.1155/2022/9911982

**Published:** 2022-01-04

**Authors:** Yi Zhang, Yong Tang, Jianhua Yan

**Affiliations:** Xinhua Hospital, School of Medicine, Shanghai Jiao Tong University, Shanghai, China

## Abstract

Long noncoding RNAs (lncRNAs) are untranslated transcripts greater than 200 nucleotides in length. Despite not being translated, they play a role in the regulation of transcription, translation, and other cellular processes and have been identified as key regulator in the progression of atherosclerosis. This study focused on the lncRNA X-inactive specific transcript (XIST), which participates in the regulation of X chromosome inactivation. XIST is produced by the *XIST* gene and is located on human chromosome Xql3.2. We also focused on discovering the possible role and mechanism of lncRNA XIST in oxidized low-density lipoprotein- (ox-LDL-) stimulated vascular smooth muscle cells (VSMCs), which could further help evalute its possible a role in the progression of atherosclerosis. XIST was overexpressed in ox-LDL-stimulated VSMCs, while the expression of miR-539-5p was decreased. XIST knockdown hindered the proliferation and migration of ox-LDL-treated VSMCs. XIST inhibits the miR-539-5p expression through direct interaction. Besides, miR-539-5p inhibitors can partially reverse the effect of XIST depletion on the proliferation and migration of VSMCs induced by ox-LDL stimulation. Further mechanistic analysis showed that secreted phosphoprotein 1 (*SPP1*) is the target of miR-539-5p, and XIST acts as a competing endogenous RNA for miR-539-5p to enhance the expression of *SPP1*. In addition, miR-539-5p inhibitor exerts its proliferation and migration effects by activating the miR-539-5p/SPP1 axis in VSMCs stimulated by ox-LDL. In conclusion, our study findings show that XIST inhibition can inhibit the proliferation and migration of atherosclerosis vascular smooth muscle cells, which provides a new theoretical basis for atherosclerosis treatment.

## 1. Introduction

Atherosclerosis is one of the most common vascular diseases worldwide and can lead to stroke and coronary heart disease. Atherosclerosis and its associated health risks are a major cause of death worldwide [1–3]. The adaptive immune response is triggered by the presentation of antigens, which in atherosclerosis are often considered to modify the (new) epitopes of LDL. Oxidized low-density lipoprotein (ox-LDL) is a crucial risk factor for atherosclerosis progression and affects the proliferation and invasion of vascular smooth muscle cells (VSMCs) [4].

Over the past decade, it has become increasingly clear that a number of epigenetic pathways control the differentiation and activation of immune cells. Epigenetic mechanisms, including DNA methylation, histone modification, and noncoding RNAs, have a significant and widespread effect on the gene expression. Chromatin modifiers, such as histone deacetylase (HDAC) and histone methyltransferase (HMT), have been found to modify the lipid metabolism and inflammatory responses in macrophages after exposure to ox-LDL [5]. For example, a myeloid-specific deletion of HDAC3 leads to the production of a large anti-inflammatory macrophage phenotype of TGF-*β*, which induces collagen production and fibrous cap formation in smooth muscle cell atherosclerosis models *i*n vivo [6]. Interestingly, when monocytes and macrophages were exposed to ox-LDL or hypercholesterolemia in vivo, differentiated macrophages showed a higher inflammatory state than the unexposed controls. This posttraining adaptive immune response can be reversed by preconditioning with the methyltransferase inhibitor methyl-adenosine, suggesting an essential role for epigenetic histone modifications in this process [7].

In addition, other epigenetic modulators, such as long noncoding RNAs (lncRNAs) [8] and microRNAs [9] can be regulated after lipid loading in monocytes/macrophages. LncRNAs are defined as a class of novel transcripts, over 200 nucleotides in length, usually lack the ability to encode proteins, and were once regarded as transcriptional noise [10]. They can exert their regulatory functions by recruiting or sponging miRNAs. LncRNAs, as gene regulators, play a crucial role in a variety of diseases (e.g., inflammation [11] and immune response [12]). LncRNA X-inactive specific transcript (XIST) is a 17 kb long RNA transcribed by the inactivated X chromosome. It is believed to be associated with X chromosome inactivation in female mammals, providing a compensation for the imbalance of X-linked gene dosage between the sexes [13, 14]. It has been reported that lncRNA XIST plays an essential role in vascular diseases, such as promoting myocardial infarction through miR-101a-3p-targeted FOS regulation [15]. Oxygen-glucose deprivation (OGD) induces the lncRNA XIST expression, and in turn, the overexpression of lncRNA XIST promotes OGD-Induced neuronal injury [16]. LncRNA XIST knockdown was found to inhibit the ox-LDL-stimulated endothelial cell inflammatory response and apoptosis by increasing the expression of target miRNAs [17, 18].

A potential target miRNA of lncRNA XIST, as indicated by several starbase maps, is miR-539-5p, located on chromosome 14q32.31. MiR-539-5p is a novel tumor suppressor in multiple cancers and has been reported by various studies [19, 20]. The role of lncRNA XIST in the proliferation and infiltration of VSMCs in atherosclerosis is still not fully understood, and further research is required to understand this process.

In this study, we aimed to further explore the potential roles and their accompanying molecular basis of XIST in the process of VSMC proliferation and infiltration to discover potential therapeutic targets for atherosclerosis.

## 2. Materials and Methods

### 2.1. Animals

The process is conducted on the basis of National Institutes of Health (NIH) Animal Use Guidelines. All C57BL/6 mice (5-6 weeks) were purchased Sipeifu Biotechnology Co., Ltd. (Beijing, China). Mice were randomly divided into two groups: the normal diet group (Ctrl, 14.7 kJ/g, 13% of energy from fat, 20% from protein, and 67% from carbohydrate, *n* = 6) and the high-fat diet group (HFD, 21.8 kJ/g, 60% of energy from fat, 20% from protein, and 20% from carbohydrate, *n* = 18). All mice were fed for 24 weeks in a standard SPF environment with a 12/12 light-dark cycle at 20-25°C to induce atherosclerosis. For silencing XIST, the negative control shRNA (sh-NC) lentivirus and XIST shRNA (sh-XIST) lentivirus were constructed by Genechem Biotechnology Company (Genechem, Shanghai, China). The mice were injected via tail vein with a single dose (50ul/g) of lentiviruses expressing sh-XIST (1 × 10^9^ PFU) or sh-NC every four weeks. All conducted experiments were approved by the ethic committee of Shanghai Jiao Tong University. The procedures were performed according to the guideline of Guide for the Care and Use of Laboratory Animals (NIH Publications, 1996).

### 2.2. Cell Line Culture and Transfection

The cell lines were conventionally cultured in complete medium containing 10% fetal bovine serum and 1% penicillin/streptomycin (Gibco BRL). To observe the effect of ox-LDL on XIST and miR-539-5p expression, 0, 25, 50, and 100 *μ*g/mL gradient doses of ox-LDL were added to VSMCs for 24 h. XIST short hairpin RNA (shRNA), miR-539-5p mimic, and the corresponding control were purchased from RiboBio Co., Ltd. (Guangzhou, China). VSMC transfection was performed using Lipofectamine 2000 (Invitrogen, Carlsbad, CA, USA). For the rescue experiment, VSMCs were cotransfected with 1 *μ*g recombinant lentivirus vector expressing secreted phosphoprotein 1 (SPP1) or empty lentivirus vector. VSMCs cotransfected with the lentivirus vector and control mimics were used as control groups.

### 2.3. RNA Extraction and Real-Time Quantitative PCR Assays

Target miRNAs were extracted from mouse aortic tissue and VSMCs using the miRNA Isolation Kit (Invitrogen). Total RNA was isolated using TRIzol (Invitrogen) in line with the manufacturer's instructions. A 7300 real-time PCR system (Applied Biosystem, Foster City, CA, USA) was used to amplify target genes. U6 normalized the expression of miR-539-5p and *β*-actin was used as an internal control for mRNA expression. The primers used in this study were as follows: XIST (forward): 5′-ACGCTGCATGTGTCCTTAG-3′, (reverse): 5′-GAGCCTCTTATAAGCTGTTTG-3′; miR-539-5p: 5′-GGAGAAAUUAUCCUUGGUGUGU-3′; *SPP1* (forward): 5′-TTTGTTGTAAAGCTGCTTTTCCTC-3′, (reverse): 5′-GAATTGCAGTGATTTGCTTTTGC-3′; *U6* (forward): 5′-CTCGCTTCGGCAGCACA-3′, (reverse): 5′-AACGCTTCACGAATTTGCGT-3′; *β*-actin (forward): 5′-ATCACTGCCACCCAGAAGAC-3′, (reverse): 5′-TTTCTAGACGGCAGGTCAGG-3′.

### 2.4. Biochemical Analysis

The serum of the mice was collected by retroorbital and measured for triglyceride (TG), high-density lipoprotein cholesterol (HDL-C), and low-density lipoprotein cholesterol (LDL-C) levels according to the manufacturer's instructions. The concentrations of HDL-C, LDL-C, and TG were determined by an automatic biochemical analyzer Olympus AU2700 (Olympus, Tokyo, Japan).

### 2.5. Histological Analysis

The same portion of the aorta samples in mice was used for histopathological examination. Tissues were fixed in 4% paraformaldehyde, embedded in paraffin, cut into 5 *μ*m sections, and then stained with hematoxylin and eosin (H&E). The degree of aorta damage was estimated by an investigator blinded to the experiment as previously described [21].

### 2.6. Masson Staining

The block embedded in OCT was cut to a thickness of 5 *μ*m. The slices were stained with Masson dye solution. Masson staining demonstrates the presence of irregular atherosclerotic plaques in blood vessels. Collagenous fibers in the vessel wall and inside and outside the plaques are green, and smooth muscle cells are red.

### 2.7. CCK-8 Assays

The target cells were seeded into 96-well plates with a cell density of 2 × 10^3^ in each well. Each group is provided with three repeat holes. CCK-8 was determined as follows: 10 *μ*L Cell Counting Kit 8 (Dojindo, Kumamoto, Japan) was added to the medium of 100 *μ*L DMEM per well and cocultured in the dark at 37°C for 2 h. The absorbance was measured by a microplate reader (Bio-Rad, Hercules, CA) at a wavelength of 450 nm.

### 2.8. Wound Healing Assay

Wound healing tests were used to evaluate cell migration in VSMCs. Cells are inoculated to produce confluent monolayers in a 6-well plate. The fused cells were scraped with 200 *μ*l sterile pipette aspirator to cause the wound. After 48 hours of culture, the wound caused by the scratches was evaluated using a microscope. The image represents one of at least three repeated experiments.

### 2.9. Transwell Invasion Assay

In a 24-well transwell chamber (Corning Incorporation, Corning was used to determining the cell invasion, NY, USA), VSMCs were seeded into the upper chamber at a 4 × 10^4^ cell density and leached with matrigel (BD Biosciences, Franklin Lakes, NJ, USA). And the underlying cells were observed and counted under microscope (Leica) after incubation at 37°C for 24 h [22].

### 2.10. Western Blot Analysis

Use a lysate buffer with added proteases and phosphatase inhibitors (Pierce; Thermo Fisher Scientific, Inc.). Total proteins were extracted from cultured cells. Each protein sample was isolated with 10% SDS-PAGE and transferred to a nitrocellulose membrane (Pall Life Sciences, Port Washington, NY, USA). After being sealed at room temperature with 5% bovine serum albumin (Beyotime Biotechnology Institute, Haimen, China) for 1 h, the membrane and primary antibody were incubated overnight at a dilution of 1 : 1,000 under 4°C. Antibodies against matrix metalloproteinase MMP2 (sc-10736), MMP9 (sc-10737), SPP1 (ab247061), PCNA (sc-10737), and Ki67 (sc-10737) were purchased from Santa Cruz Biotechnology (CA, USA). HRP-conjugated anti-mouse immunoglobulin G at a dilution of 1 : 5,000 (ZB-2305; ZSGB-Bio) was placed at room temperature for 1 hour. Anti-*β*-actin at a dilution of 1 : 1,000 (sc -47778) was used as control.

### 2.11. Dual-Luciferase Reporter Gene System

The XIST fragment and mutant XIST fragment were cloned into pMIR Glo expression vector (Sangon Biotech, China) to produce XIST wild-type reporter vector (XIST-WT) and XIST-MUT. The above vectors miR-539-5p simulators and negative control were transfected into cells by lipid transfection amine (Invitrogen, CA, USA).

### 2.12. RNA Immunoprecipitation (RIP) Assay

RIP analysis was performed by EZ-Magna RIP kit (Millipore, Billerica, MA, USA) and Argonaute 2 antibody to investigate the presence of XIST in the RNA-induced silencing complex (RISC). In short, VSMCs were cleaved in a RIP lysis buffer and then incubated with protein A/G magnetic beads and antibodies against IgG (Millipore) or AGO2 (Abcam). The RNA from the bead-bound complex was then purified. Finally, the enrichment pattern of IgG antibody or AGO2 antibody against XIST and miR-539-5p was detected by RT-qPCR.

### 2.13. Tail Vein Metastatic Assay

A549-sh-XIST cells and A549-sh-Ctrl cells were harvested, washed twice with PBS, and suspended in PBS. A total of 6 mice per group received 5 × 10^6^ cells in 150 *μ*l PBS by tail vein injection. The mice were sacrificed at 20 weeks postinjection.

### 2.14. Bioinformatic Analysis

Starbase software (http://starbase.sysu.edu).Cn/index) was used to predict the binding region of miR-539-5p and XIST. The binding sites of miR-539-5p and SPP1 were predicted using miRDB (http://www.mirdb.org/) and ENCORI software. Coexpression analysis between SPP1 and other antioxidant related genes was performed using online STRING software (https://string-db.org/) and analyzed the generic carcinoma (http://starbase.sysu.edu.cn/panCancer.php), in order to research XIST and prognosis, miR-539-5p, and SPP1 expression level correlation. All the search program were executed using default parameters.

### 2.15. Statistical Analysis

All data were expressed as mean ± SD. Statistical significance was analyzed using SPSS 20.0 software (IBM Corporation, Armonk, NY). The Student *t*-test was used to determine the statistical differences between the two groups, and ANOVA was used to analyze the statistical differences among the multiple groups. Two tail *P* < 0.05 was considered statistically significant.

## 3. Results

### 3.1. The Expression of XIST and Cell Proliferation Is Increased in ox-LDL-Stimulated VSMCs

**Figure 1:**
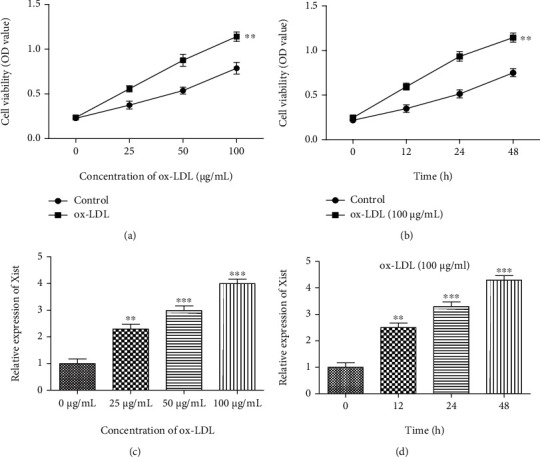
The expression of XIST is increased, and cell proliferation is promoted in ox-LDL-stimulated VSMCs. (a, b) CCK-8 assay showed the cell viability in ox-LDL stimulated VSMCs. (c, d) The expression of XIST in VSMCs by qRT-PCR, ^∗∗^*P* < 0.01, ^∗∗∗^*P* < 0.001.

CCK-8 analysis showed that ox-LDL stimulation increased cell viability in a dose- and time-dependent manner in VSMCs (Figures 1(a) and 1(b)). In addition, RT-qPCR analysis showed that the XIST expression also increased in a dose- and time-dependent manner (Figures 1(c) and 1(d)). These results show that ox-LDL has effects on the proliferation of VSMCs and XIST expression.

### 3.2. Inhibition of XIST Expression and the Effects of XIST on Atherosclerotic Mice

**Figure 2:**
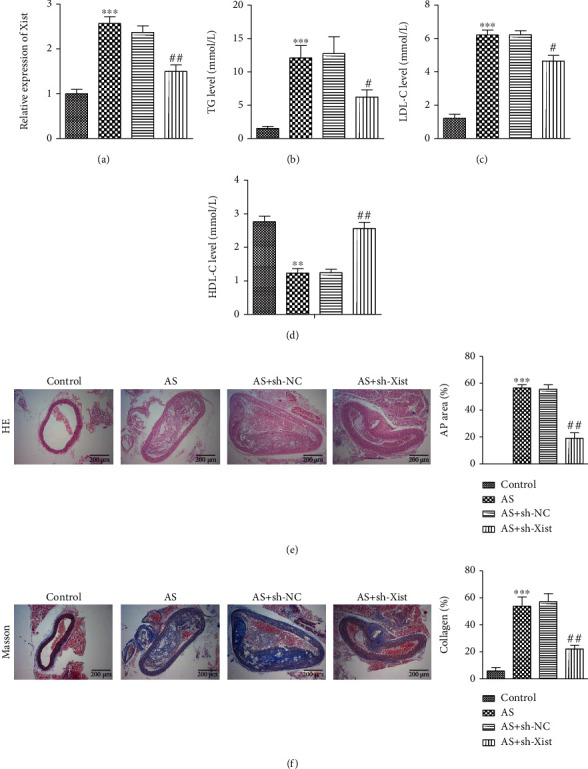
Effects of XIST on atherosclerosis mice. (a) RT-qPCR was used to detect the efficiency of knockdown XIST in mice. (b)–(d)The bar graph showed TG and LDL as well as a low HDL levels in sh-NC group vs. sh- XIST group, ^∗^*P* < 0.05, ^∗∗^*P* < 0.01. (e) HE staining indicated that the area of aortic AP in the sh-XIST group compared with other groups, bars = 200 *μ*m, ^∗∗^*P* < 0.01. (f) Masson staining showed that the collagen content in the area of aortic AP in other groups decreased relative to the normal group, bars = 200 *μ*m. ^∗∗^*P* < 0.01, ^∗∗∗^*P* < 0.001, ^#^*P* < 0.05, ^##^*P* < 0.01.

First, we interfered with the XIST expression in atherosclerotic mice (AS) through the sh-RNA of XIST to determine the role of XIST in the process of atherosclerosis. Atherosclerotic mice were injected with sh-NC or sh-XIST lentiviruses, and biochemical parameters and morphology were measured. RT-qPCR analyses confirmed that the introduction of sh-XIST inhibited the XIST expression compared to the negative control sh-RNA (sh-NC) in ox-LDL-stimulated VSMCs (Figure 2(a)). The atherosclerotic mice had extremely high triglyceride (TG) and LDL levels and low HDL levels compared to those in the control group. However, sh-XIST intervention was shown to inhibit the increase in TG and LDL levels as well as the decrease in HDL levels (Figures 2(b)–2(d)). As shown in Figure 2(e), HE staining showed a significant increase in the area of aortic atherosclerotic plaque (AP) in the atherosclerotic mice compared to the control mice. Compared with the AS + sh-NC group, the AP area of the aorta was markedly reduced in the AS + sh-XIST group. This suggests that XIST promotes the AP formation in mice with atherosclerosis. Masson staining showed that the collagen content of the aortic AP area in the other groups was reduced. There was no significant difference in collagen content of aortic plaques between the AS and the AS + sh-NC groups. However, the aortic plaques of the AS + sh-XIST group had a higher collagen content than that of the AS group, suggesting that XIST reduces the collagen content of aortic plaques in mice with atherosclerosis (Figure 2(f)).

### 3.3. Effects of XIST on Proliferation, Migration, and Invasion of ox-LDL-Stimulated VSMCs

**Figure 3:**
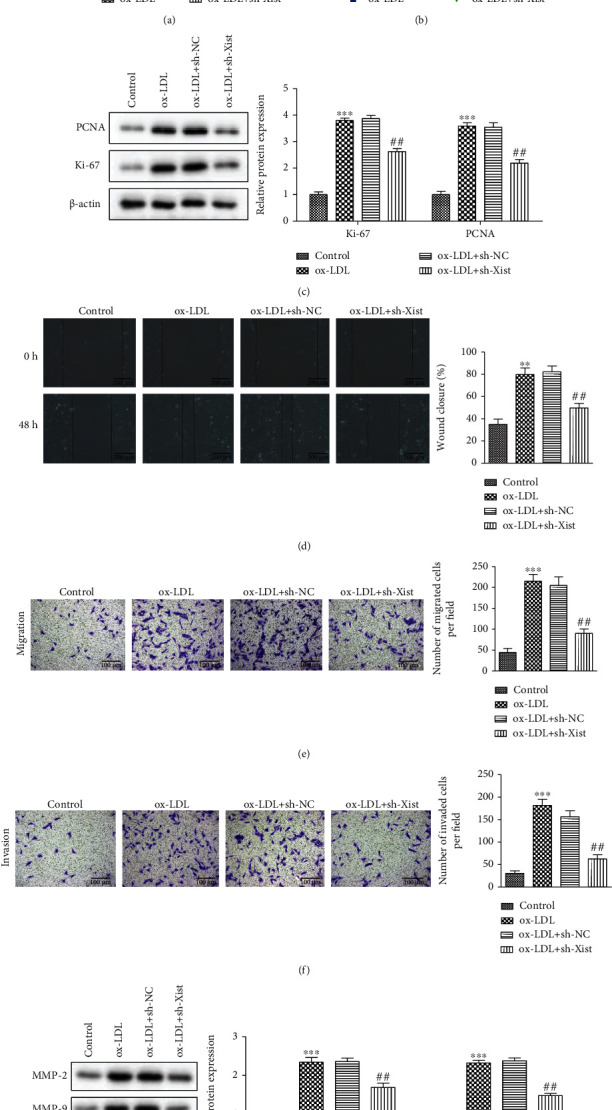
Effects of XIST on proliferation, migration, and invasion of ox-LDL- stimulated vascular smooth muscle cells. (a) RT-qPCR was used to detect the efficiency of knockdown XIST in VSMCs. (b) CCK-8 assay showed cell viability in different groups. (c) The expression of Ki67 and PCNA protein. (d)–(f) Wound-healing assay (bars = 200 *μ*m) and transwell assay (bars = 100 *μ*m) showed effects on VSMCs invasion and migration. (g) The expression of MMP-2 and MMP-9 protein detected by western blot analyses. ^∗∗^*P* < 0.01, ^∗∗∗^*P* < 0.001, ^#^*P* < 0.05, ^##^*P* < 0.01.

To further investigate the role of XIST in atherosclerosis, we used ox-LDL treatment of a human vascular smooth muscle cell line, VSMC, which has been widely used for in vitro studies of atherosclerosis. ox-LDL stimulation was found to promote the expression of XIST compared with the control group, whereas ox-LDL-VSMCs transfected with sh-XIST downregulated the expression of XIST (Figure 3(a)). The proliferation ability of VSMCs was detected using the CCK8 assay. We found that XIST knockdown reversed the effect of ox-LDL stimulation on VSMC proliferation (Figure 3(b)). Western blotting analysis showed that sh-XIST inhibited the ox-LDL-induced upregulation of proliferation-related proteins PCNA and Ki67 (Figure 3(c)). Next, we performed wound healing and transwell assays to test whether XIST had any effect on the invasion and migration of VSMCs. We found that XIST knockdown reversed ox-LDL-induced cell migration (Figures 3(d)–3(f)). In addition, the expression of migration and invasion-related proteins, including MMP-2 and MMP-9, was greatly increased in ox-LDL-VSMCs, and XIST silencing reduced ox-LDL function (Figure 3(g)). These results suggest that XIST promotes the proliferation, migration, and invasion of VSMCs, which may in turn promote the formation of atherosclerotic plaques and the overall progression of atherosclerosis.

### 3.4. Targeting the Relationship between LncRNA-XIST and miR-539-5p

**Figure 4:**
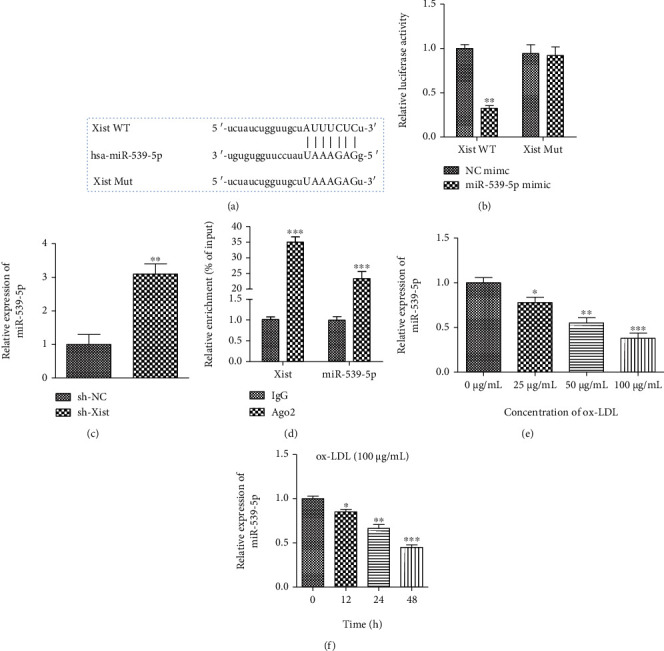
Targeting the relationship between lncRNA -XIST and miR-539-5p. (a) The relationships of base complementary pairing between XIST and miRNAs were predicted by starbase. (b) The targeting relationship between XIST and miRNAs was determined by dual luciferase report assay. (c) The bar graph showed changes of miR-539-5p expression level after downregulating XIST expression in VSMCs. (d) VSMC expressing of XIST and miR-539-5p was conducted with Ago2/RISC immunoprecipitation by Pan-Ago2 antibody, and IgG was used as a negative control. (e, f) The expression of miR-539-5p in VSMCs was in a dose- and time-dependent manner. ^∗∗^*P* < 0.01, ^∗∗∗^*P* < 0.001, ^∗^*P* < 0.05.

To further study the molecular mechanism of the role of XIST in the VSMC process, we identified potential miRNAs of targets of XIST. We found that several complementary sites existed between XIST and miR-539-5p (Figure 4(a)). Therefore, we investigated whether miR-539-5p directly interacts with XIST. We transfected the constructed XIST-WT or XIST-MUT luciferase reporter gene together with the miR-539-5p mimic or its negative control (NC) into VSMCs. Subsequent luciferase assays showed that the introduction of miR-539-5p mimic resulted in greater than 50% loss of luciferase activity in XIST-WT reporter genes compared to the NC. However, because the putative binding site between XIST and miR-539-5p was mutated, miR-539-5p did not affect the luciferase activity of the XIST-MUT reporter gene (Figure 4(b)). As shown in Figure 4(c), sh-XIST transfection resulted in a significant increase in the miR-539-5p expression. To validate the direct binding of XIST and miR-539-5p, an AGO2 antibody was used for RIP analysis to determine whether XIST and miR-539-5 pp were involved in RISC. As shown in Figure 4(d), compared with the control IgG antibody, AGO2 antibody-enriched XIST, and miR-539-5p, indicating the presence of XIST and miR-539-5p in the RISC. These data suggest that XIST and miR-539-5p bind directly to each other in VSMCs. RT-qPCR results showed that a dose- and time-dependent ox-LDL treatment resulted in a decrease in the miR-539-5p expression in VSMCs (Figures 4(e) and 4(f)). Together, these results suggest that miR-539-5p mediates the anti-proliferative effect of XIST knockdown in ox-LDL-stimulated VSMCs.

### 3.5. Effects of the miR-539-5p on Proliferation, Migration, and Invasion of ox-LDL Stimulated VSMCs

**Figure 5:**
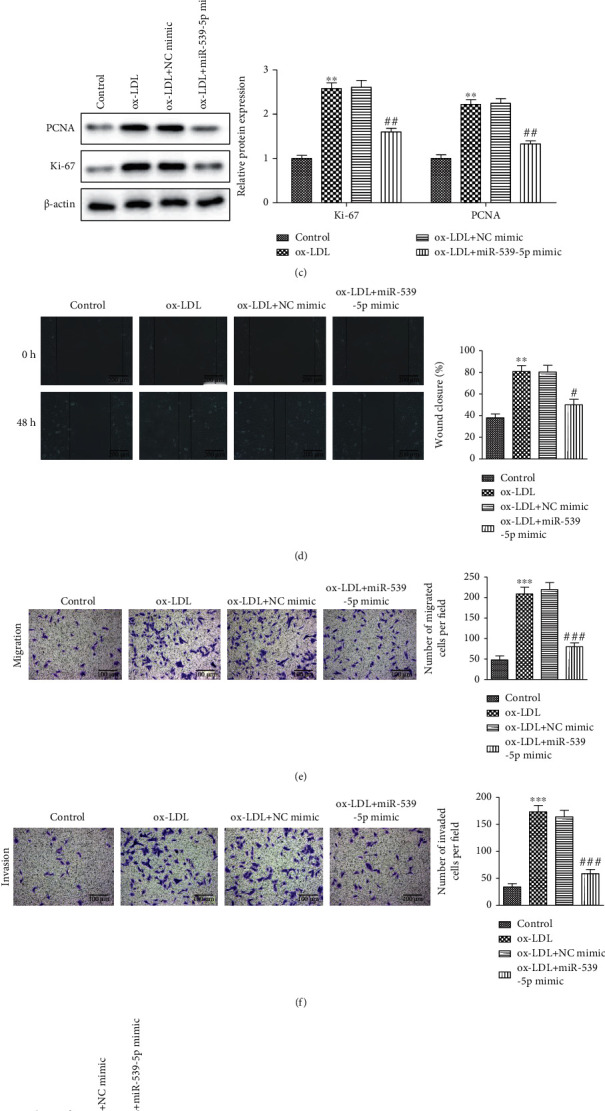
Effects of miR-539-5p on proliferation, migration, and invasion of ox-LDL stimulated VSMCs. (a) RT-qPCR was used to detect the efficiency of overexpression miR-539-5p in VSMCs. (b) The cell viability of VSMCs after overexpression of miR-539-5p was detected by CCK-8 assay in NC mimic group vs. miR-539-5p mimic group. (c) The expression levels of Ki67 and PCNA protein. (d)–(f) Wound-healing assay (bars = 200 *μ*m) and transwell assay (bars = 200 *μ*m) showed the effects on invasion and migration of VSMCs. The bar graph showed the percentage of cells in each group. (g) The expression of MMP-2 and MMP-9 protein detected by western blot analyses. ^∗∗^*P* < 0.01, ^∗∗∗^*P* < 0.001, ^#^*P* < 0.05, ^##^*P* < 0.01, ^###^*P* < 0.001.

Ox-LDL-stimulated VSMCs showed lower miR-539-5p levels than the untreated cells. In the presence of ox-LDL stimulation, cells transfected with a control mimic showed similar levels of miR539-5p, while transfection of the miR-539-5p mimic increased overall miR-539-5p expression (Figure 5(a)). ox-LDL treatment was found to promote cell activity, while the overexpression of miR-539-5p resulted in a significant decrease in cell activity in ox-LDL-stimulated VSMCs (Figure 5(b)). The upregulation of PCNA and Ki67 induced by ox-LDL was partially inhibited by miR-539-5p mimics (Figure 5(c)). These findings suggest that miR-539-5p has antiproliferative effects in vitro. The migration ability of VSMCs was determined using wound healing and transwell assays to identify the effect of the miR-539-5p expression on VSMC migration. The results showed that cells in the ox-LDL + miR-539-5p mimic group migrated more slowly than those in the ox-LDL + NC mimetic group and were unable to heal the cut wound (Figure 5(d) and 5(e)). In addition, a crosspore invasion assay showed that, compared with the ox-LDL + NC group, the invasion ability of VSMCs in the ox-LDL + miR-539-5p group was reduced (Figure 5(f)). Western blotting analysis showed that overexpression of miR-539-5p inhibited the ox-LDL-induced upregulation of MMP-2 and MMP-9 in VSMCs (Figure 5(g)). These results confirmed that miR-539-5p inhibited the proliferation, migration, and invasion of ox-LDL-stimulated VSMCs, which may slow down the progression of atherosclerosis.

### 3.6. Targeting the Relationship between miR-539-5p and SPP1

**Figure 6:**
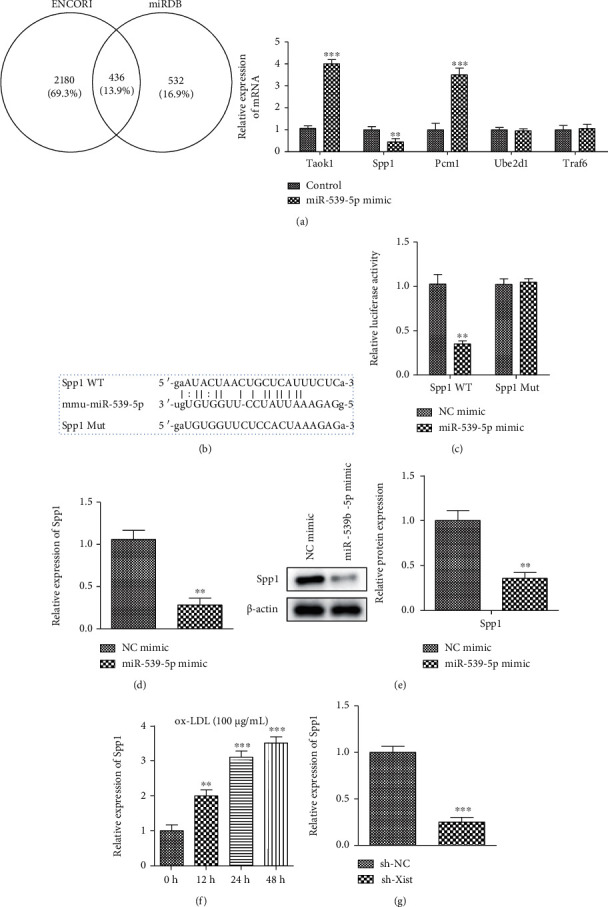
Targeting the relationship between miR-539-5p and SPP1. (a) The possible downstream targets of miR-539-5p (ENCORI and miRDB) by kinds of silica gel prediction algorithms. The mRNA expression levels of relative target genes in cells. (b) The binding sites of miR-539-5p and SPP1 were predicted by a bioinformatics website. (c) The targeting relationship between miR-539-5p and SPP1 was determined by dual luciferase report assay. (d) The bar graph showed changes of SPP1 mRNA expression level in NC mimic group vs. miR-539-5p mimic group in VSMCs, ^∗∗^*P* < 0.01. (e) Western blot analysis showed the protein levels of SPP1 in NC mimic group vs. miR-539-5p mimic group in VSMCs. (f, g) The mRNA expression level of SPP1 in VSMCs in a dose- and time-dependent manner of ox-LDL, ^∗∗^*P* < 0.01, ^∗∗∗^*P* < 0.001.

Bioinformatic analysis on Encori and miRDB predicted that *SPP1*gene was the target gene for miR-539-5p, with a putative binding site (Figures 6(a) and 6(b)). Dual-luciferase reporter assays indicated the luciferase activity in SPP1-WT cells transfec ted with miR-539-5p mimics was lower than that in the control mimics (Figure 6(c)). Next, we then detected the expression level of mRNA and protein of SPP1 in VSMC of wild-type and overexpressed miR-539-5p, and we found that the expression of SPP1 was downregulated by miR-539-5p induction (Figure 6(d) and 6(e)). In addition, SPP1 levels increased in a time-dependent manner after ox-LDL stimulation and the resulting miR-539-5p downregulation (Figure 6(f)). Sh-XIST resulted in a decrease in SPP1 expression in VSMCs (Figure 6(g)).

### 3.7. XIST/miR-539-5p/SPP1 Axis on the Proliferation, Migration, and Invasion of ox-LDL Stimulated VSMCs

**Figure 7:**
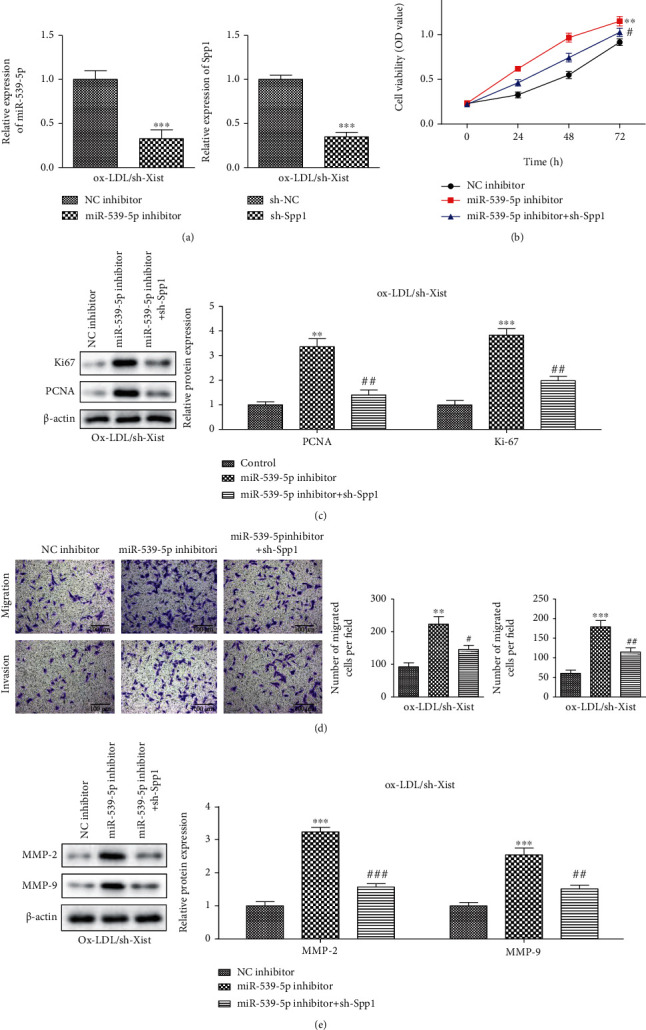
XIST/miR-539-5p/SPP1 axis on the proliferation, migration, and invasion of ox-LDL stimulated VSMCs. (a) RT-qPCR was used to detect the expression of miR-539-5p and SPP1 in VSMCs with down-regulating of XIST in each group. (b) The diagram showed cell viability of VSMCs in each group. (c) Western blot analysis showed the protein levels of Ki67 and PCNA in SPP1 inhibitors group vs. miR-539-5p inhibitors group. (d) The migration and invasion were detected by transwell assay. The bar graphs showed the percentage of migration and invasion cells in each group (bars = 200 *μ*m). (e) Western blot analysis showed the protein levels of MMP-2 and MMP-9 in NC inhibitors+ SPP1 group vs. miR-539-5p inhibitors+ SPP1 group in ox-LDL-induced VSMCs. ^∗∗^*P* < 0.01, ^∗∗∗^*P* < 0.001, ^#^*P* < 0.05, ^##^*P* < 0.01, ^###^*P* < 0.001.

We manipulated the expression of miR-539-5p and SPP1 with miR-539-5p inhibitor and sh-SPP1, respectively, and measured their knockdown efficiency with RT-qPCR (Figure 7(a)). CCK-8 analysis showed that sh-SPP1 blocked the activation of ox-LDL sh-XIST cell proliferation induced by the miR-539-5p inhibitor (Figure 7(b)), while western blotting showed that SPP1 knockout reversed ox-LDL/sh-XIST cell proliferation. The miR-539-5p inhibitors and sh-SPP1 decreased the expression of PCNA and Ki67 in ox-LDL/sh-XIST cells (Figure 7(c)). In addition, transwell analysis showed that the migration and invasion ability of ox-LDL/sh-XIST cells decreased when these cells were cotransfected with the miR-539-5p inhibitor. Sh-SPP1 reversed this reduction in migration and invasion (Figure 7(d)). Western blotting results showed that the expression of MMP-2 and MMP-9 were inhibited by the miR-539-5p inhibitors and sh-SPP1 in ox-LDL/sh-XIST cells (Figure 7(e)). Based on the results, we concluded that XIST regulates the progression of atherosclerosis through the miR-539-5p/SPP1 axis.

## 4. Discussion

Increasing evidence has shown that noncoding RNAs, including lncRNAs and miRNAs, play a crucial role in the development of atherosclerosis [23]. Previous studies on atherosclerosis have shown that there are a variety of lncRNAs, including MALAT1 [24], H19 [25], ATB [26], LEF1-AS1 [27], and ZEB1-AS1 [28], which are promising therapeutic targets. However, the biological role and mechanism of XIST in atherosclerosis remain unclear.

Little is known about the biological role and mechanism of XIST in atherosclerosis. To further investigate the molecular mechanism of XIST, the miRcode online website was used to identify its potential targets. The results showed that the predicted target miR-539-5p may interact with XIST. ox-LDL induces atherosclerosis through various mechanisms [29, 30]. We first demonstrated and then further verified that, in ox-LDL-stimulated VSMCs, the expression level of XIST increases and that of miR-539-5p decreases. Functional loss experiments showed that XIST knockdown inhibited the proliferation and migration of ox-LDL-stimulated VSMCs. Bioinformatic analysis, luciferase assay, RIP, and RT-qPCR assays further demonstrated that XIST binds to miR-539-5p to interfere with its expression. As would be expected from this, we found that the miR-539-5p expression negatively correlated with XIST expression in ox-LDL-stimulated VSMCs.

Therefore, we investigated whether miR-539-5p mediates the effects of XIST on proliferation and migration in ox-LDL-stimulated VSMCs. The results showed that in ox-LDL-stimulated VSMCs, the downregulation of miR-539-5p disrupted the antiproliferative and antimigratory effects mediated by XIST knockdown. We used Encori and miRDB to identify the potential target mRNAs of miR-539-5p and found that *SPP1* was a target as verified by luciferase assays. We found that in ox-LDL-stimulated VSMCs, the mRNA expression of *SPP1* positively correlated with the expression of the XIST expression, but negatively correlated with the expression of miR-539-5p. Our study further showed that XIST could be used as a competing endogenous RNA for miR-539-5p, to enhance the expression of the target gene *SPP1* in VSMCs.

SPP1, the former osteopontin gene, is composed of seven exons located in the 4q22.1 chromosome region [31]. Its protein product is osteopontin (OPN), which binds to a variety of integrin receptors and affects cell adhesion, migration, and survival [32]. OPN is expressed in a variety of immune cells and has been reported to act as an immune regulator, promoting cell recruitment to inflammatory sites [32–34]. Evidence from several genetic mouse models and basic studies suggest that the SPP1 gene influences the atherosclerotic process [35, 36]. According to these reports, SPP1 (OPN) gene polymorphism has been shown to affect its transcriptional activity [37]. We found that miR-539-5p deficiency induced the promotion of ox-LDL-stimulated VSMC proliferation and migration by regulating the SPP1 expression.

In conclusion, our data show that XIST promotes the proliferation and migration of ox-LDL-stimulated VSMCs through the miR-539-5p/SPP1 axis, suggesting that XIST has a key role in the progression of atherosclerosis and is a promising target for the development of effective therapies for atherosclerosis.

## Data Availability

The data underlying the findings of the paper are publicly available.
